# Supplementary material for: Low elevation of Svalbard glaciers drives high mass loss variability
Source: Nat Commun. 2020 Sep 14;11:4597. doi: 10.1038/s41467-020-18356-1 (PMC7490702; doi:10.1038/s41467-020-18356-1)
Supplement: Supplementary file 1 — Supplementary Information [file 41467_2020_18356_MOESM1_ESM.pdf]

Supplementary Information: "Low elevation of Svalbard glaciers drives high mass loss variability"

Brice Noël*[1], C. L. Jakobs[1], W. J. J. van Pelt[2], S. Lhermitte[3], B. Wouters[1,3], J. Kohler[4], J. O. Hagen[5], B. Luks[6], C. H. Reijmer[1], W. J. van de Berg[1], & M. R. van den Broeke[1]

[1]*Institute for Marine and Atmospheric research Utrecht, Utrecht University, 3584 CC Utrecht, Netherlands.*
[2]*Department of Earth Sciences, Uppsala University, SE 75236 Uppsala, Sweden.*
[3]*Department of Geoscience & Remote Sensing, Delft University of Technology, 2600 AA Delft, Netherlands.*
[4]*Norwegian Polar Institute, N-9296 Tromsø, Norway.*
[5]*Department of Geosciences, University of Oslo, 0371 Oslo, Norway.*
[6]*Institute of Geophysics, Polish Academy of Sciences, 01-452 Warsaw, Poland.*

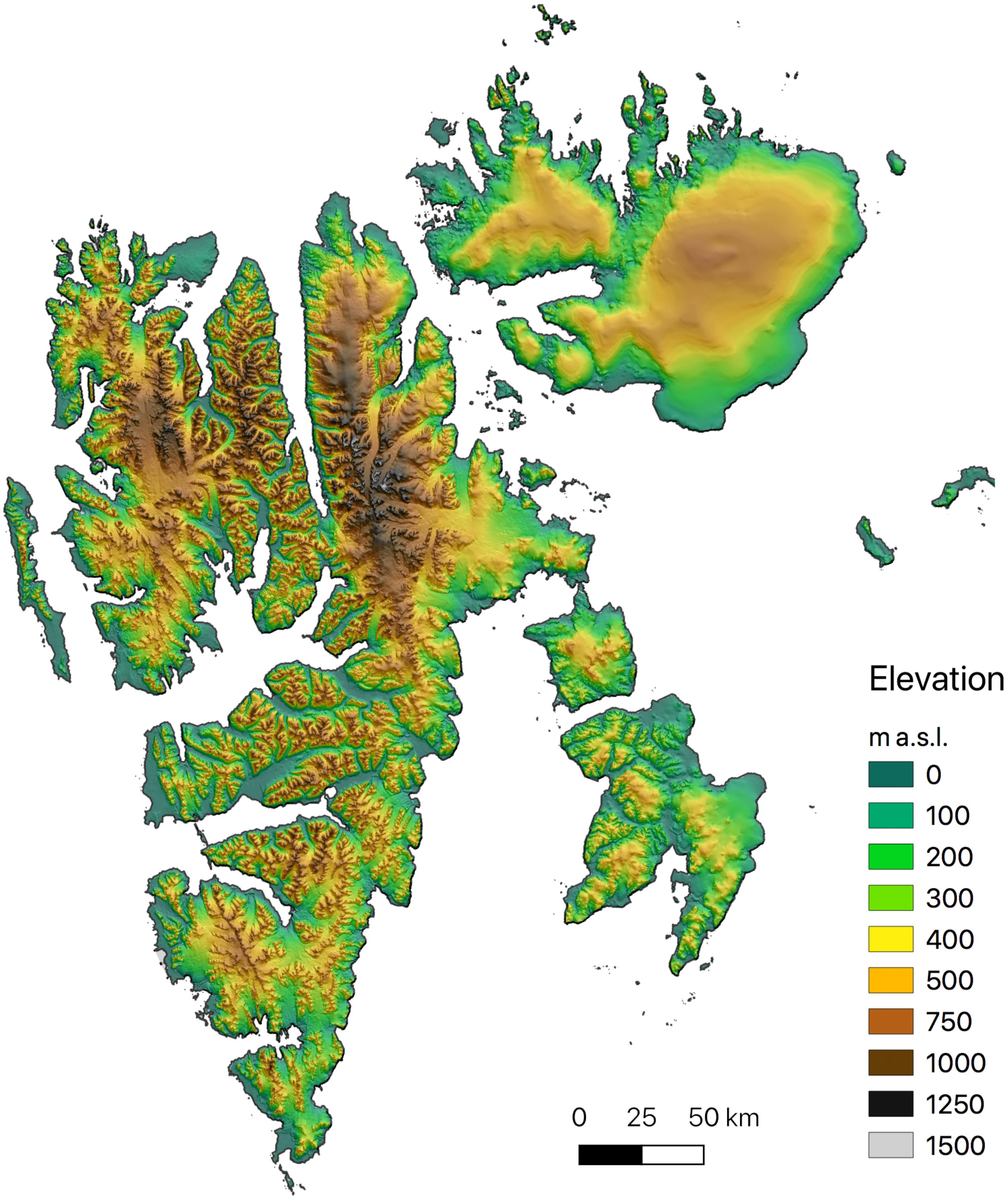

Supplementary Figure 1: **Topography of the Svalbard archipelago** Surface elevation (m a.s.l.) derived from the S0 Terreng DEM of Svalbard at 20 m spatial resolution (Norwegian Polar Institute) and down-sampled to a 500 m grid.

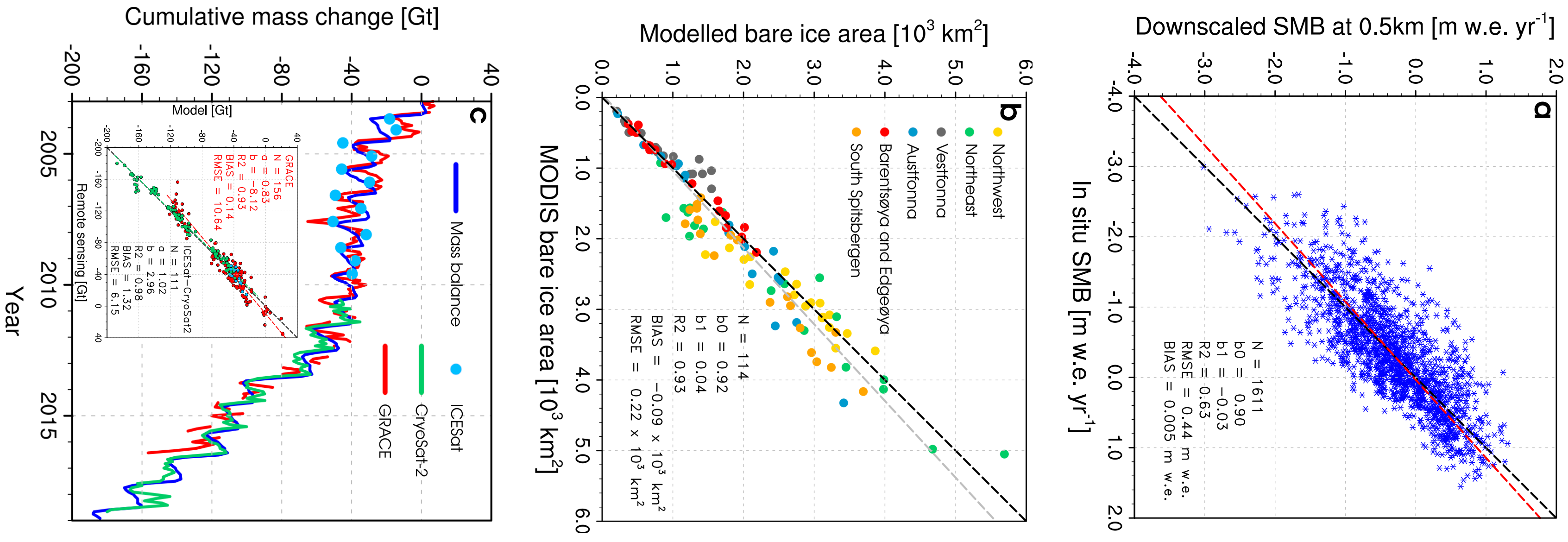

Supplementary Figure 2: **Model evaluation using in situ and remote sensing measurements** (a) Comparison between modelled and observed SMB at 101 stakes (Fig. 1a). The red dashed line represents the regression including all measurements. (b) Comparison between modelled and observed bare ice area for individual sectors. The grey dashed line corresponds to the regression using all measurements. (c) Time series of monthly cumulative reconstructed mass balance (MB = SMB minus solid ice discharge) overlapping the satellite period (2003-2018): GRACE (2003-2016), ICESat (2003-2009) and CryoSat-2 (2010-2018). The inset in Supplementary Fig. 2c shows the comparison between modelled and remotely sensed monthly cumulative mass change from GRACE, ICESat and CryoSat-2. Regressions are shown as dashed red (GRACE) and green (ICESat/CryoSat-2) line. Statistics including the number of observations (N), slope (b0) and intercept (b1) of the regression line, coefficient of determination (R^2), RMSE and mean bias between model and observations are also listed.

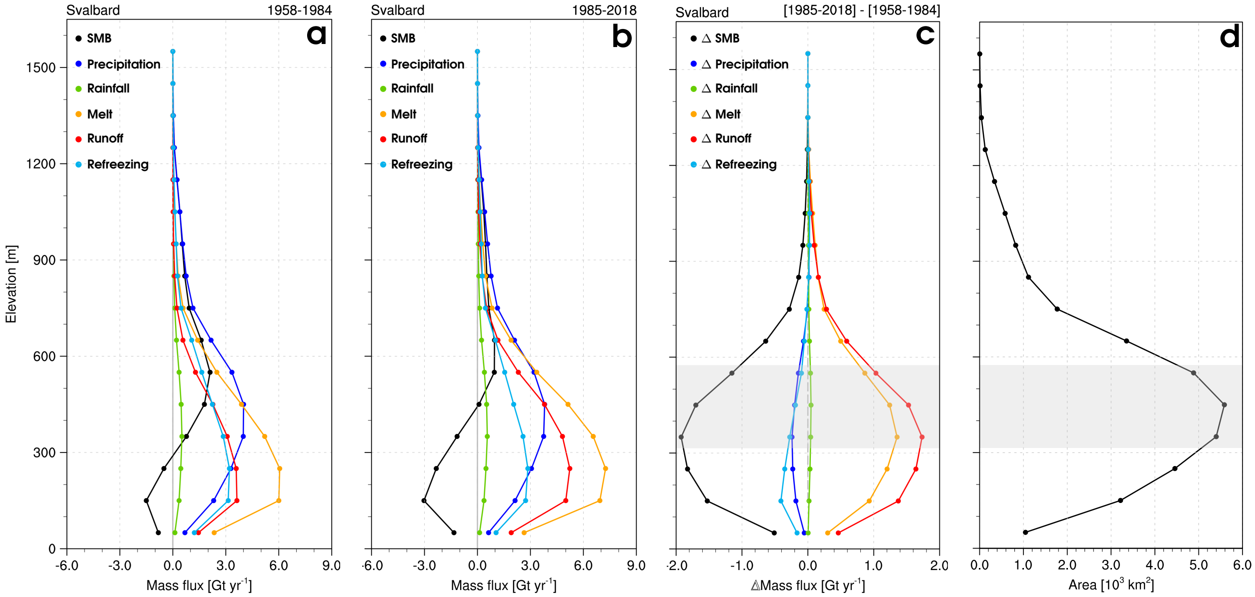

Supplementary Figure 3: **Upward migration of the firn line** Vertical profile of integrated SMB and components including total precipitation (snowfall and rainfall), rainfall, melt, runoff and refreezing for the periods (a) 1958-1984, (b) 1985-2018, and (c) the difference between the two periods (1985-2018 minus 1958-1984). (d) Hypsometry of Svalbard ice caps, i.e. integrated ice-covered area within 100 m elevation bins. The grey band spans the minimum and maximum ELA (SMB = 0) of individual sectors for the periods 1958-1984 and 1985-2018.

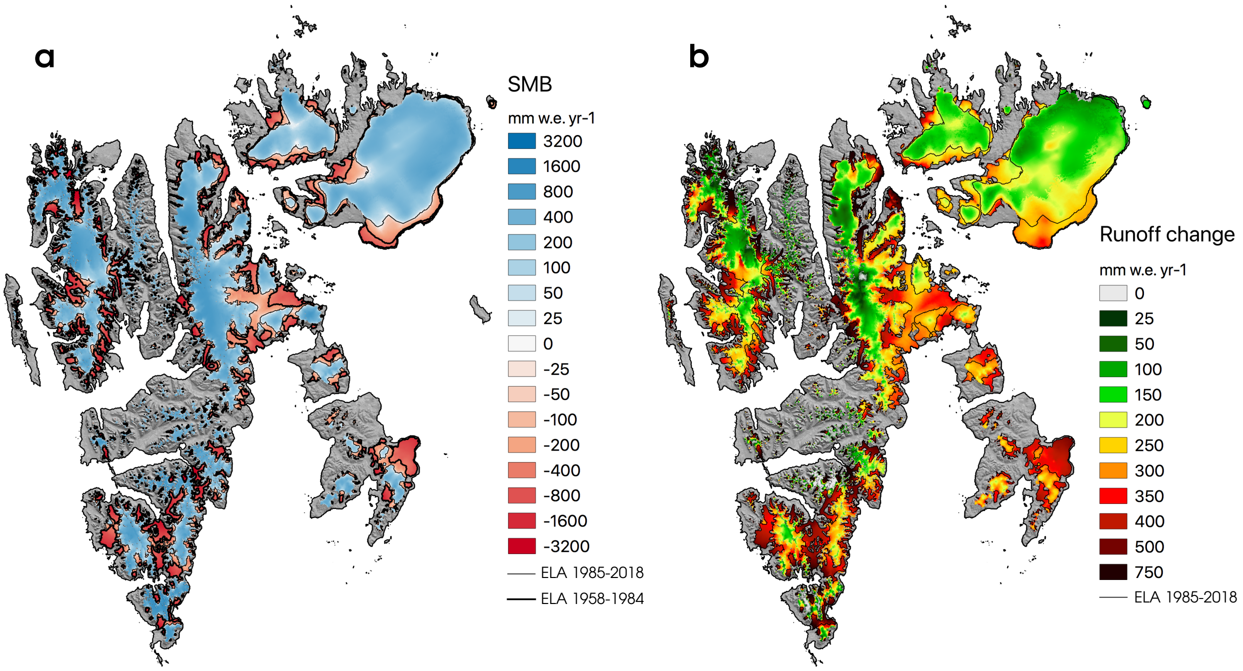

Supplementary Figure 4: **Ablation zone expansion and runoff change** (a) SMB of Svalbard ice caps averaged for the period 1985-2018. The thick and thin black lines outline the ELA (local SMB = 0) for periods 1958-1984 and 1985-2018. (b) Post-1985 change in meltwater runoff (1985-2018 minus 1958-1984). The black line outlines the 1985-2018 ELA.

Supplementary Table 1: **Model evaluation using recent mass change estimates** Comparison between mass balance (MB = SMB minus D) from the current study and previous geodetic, GRACE, model and observation-based mass change estimates. Models include the Weather Research and Forecasting model (WRF), the Modèle Atmosphérique Régional (MAR), a Positive Degree Day (PDD) and two Energy Balance Models (EBM). In our study, solid ice discharge (D) is derived from Ref. [1] before 2012 and combined Refs. [1,2] afterwards.

References	Method	Period	Units	Estimate	This study
Moholdt et al. (2010)[3]	Geodetic	2003-2008	Gt yr^{-1}	-4.1 ± 1.4	-7.1 ± 3.4
Zemp et al. (2019)[4]	Geodetic	2006-2016	Gt yr^{-1}	-16.0 ± 8.0	-9.7 ± 3.4
Wouters et al. (2008)[5]	GRACE	2003-2008	Gt yr^{-1}	-8.8 ± 3.0	-7.1 ± 3.4
Mèmin et al. (2011)[6]	GRACE	2003-2009	Gt yr^{-1}	-9.1 ± 1.0	-6.0 ± 3.4
Gardner et al. (2013)[7]	GRACE	2003-2009	Gt yr^{-1}	-6.8 ± 2.0	-6.0 ± 3.4
Jacob et al. (2012)[8]	GRACE	2003-2010	Gt yr^{-1}	-3.0 ± 2.0	-5.7 ± 3.4
Matsuo et al. (2013)[9]	GRACE	2004-2008	Gt yr^{-1}	-6.8 ± 3.7	-3.6 ± 3.4
Matsuo et al. (2013)[9]	GRACE	2004-2012	Gt yr^{-1}	-3.7 ± 3.0	-4.7 ± 3.4
Wouters et al. (2019)[10]	GRACE	2002-2016	Gt yr^{-1}	-7.2 ± 1.4	-9.3 ± 3.4
Aas et al. (2016)[11]	WRF	2003-2013	Gt yr^{-1}	-8.7	-9.1 ± 3.4
Lang et al. (2015)[12]	MAR	1979-2013	Gt yr^{-1}	-8.4	-8.0 ± 3.4
Möller et al. (2018)[13]	PDD	1957-2010	Gt yr^{-1}	1.0	-4.1 ± 3.4
Østby et al. (2017)[14]	EBM	1957-2014	Gt yr^{-1}	-4.0	-5.0 ± 3.4
Van Pelt et al. (2019)[15]	EBM	1957-2018	Gt yr^{-1}	-3.0	-5.9 ± 3.4
Schuler et al. (2020)[16]	Data upscaling	2000-2019	Gt yr^{-1}	-8.0 ± 6	-11.4 ± 3.4

Supplementary Table 2: **State of Svalbard glaciers pre-1985** This table lists the mass balance (MB = SMB minus D), SMB and components, firn refreezing capacity, ablation zone area (i.e. as a fraction of the total glacier area), and ELA (SMB = 0) for individual sectors and the whole of Svalbard averaged over the period 1958-1984. Solid ice discharge (D) is estimated from Ref. [1]. ELA uncertainty is estimated as one standard deviation of the period 1958-1984.

1958-1984	Units	NW	NE	VF	AF	BE	SS	Svalbard
MB	Gt yr^{-1}	-	-	-	-	-	-	-0.4 ± 3.4
SMB	Gt yr^{-1}	0.3 ± 0.3	2.0 ± 0.4	0.5 ± 0.1	2.9 ± 0.3	0.2 ± 0.1	0.6 ± 0.3	6.3 ± 1.6
Precipitation	Gt yr^{-1}	4.2	5.5	1.4	5.1	1.6	4.8	23.0
Runoff	Gt yr^{-1}	3.9	3.5	0.9	2.1	1.5	4.2	16.3
Melt	Gt yr^{-1}	7.4	6.8	1.5	4.3	1.9	6.4	28.7
Refreezing	Gt yr^{-1}	4.5	4.1	0.8	2.7	0.7	3.4	16.5
Refreezing capacity	%	57.6	58.7	52.1	60.3	37.2	48.9	54.4
Ablation zone area	%	35.4	25.1	21.5	11.6	36.0	34.8	27.1
ELA	m	470 ± 65	380 ± 100	270 ± 84	220 ± 60	280 ± 80	340 ± 50	350 ± 60

Supplementary Table 3: **State of Svalbard glaciers post-1985** This table lists the mass balance (MB = SMB minus D), SMB and components, firn refreezing capacity, ablation zone area (i.e. as a fraction of the total glacier area), and ELA (SMB = 0) for individual sectors and the whole of Svalbard averaged over the period 1985-2018. Solid ice discharge (D) is estimated from Ref. [1] before 2012 and combined Refs. [1,2] afterwards. ELA uncertainty is estimated as one standard deviation of the period 1985-2018.

1985-2018	Units	NW	NE	VF	AF	BE	SS	Svalbard
MB	Gt yr^{-1}	-	-	-	-	-	-	-10.2 ± 3.4
SMB	Gt yr^{-1}	-1.6 ± 0.3	-0.2 ± 0.4	0.0 ± 0.1	1.4 ± 0.3	-0.6 ± 0.1	-1.3 ± 0.3	-2.6 ± 1.6
Precipitation	Gt yr^{-1}	4.2	5.6	1.3	5.0	1.5	4.7	22.8
Runoff	Gt yr^{-1}	5.9	5.8	1.3	3.6	2.2	6.0	25.2
Melt	Gt yr^{-1}	8.9	8.6	1.8	5.5	2.5	7.6	35.4
Refreezing	Gt yr^{-1}	4.2	3.9	0.7	2.5	0.6	3.0	15.1
Refreezing capacity	%	45.0	43.3	39.2	44.8	23.9	36.1	40.6
Ablation zone area	%	49.0	43.3	41.0	27.5	61.0	51.5	43.9
ELA	m	550 ± 65	510 ± 130	360 ± 100	340 ± 110	370 ± 85	420 ± 55	440 ± 80

Supplementary References

1. Blaszczyk, M., Jania, J. & Hagen, J. O. Tidewater glaciers of Svalbard: Recent changes and estimates of calving fluxes. *Polish Polar Research* **30**, 85 – 142 (2009).

2. Dunse, T. *et al.* Glacier-surge mechanisms promoted by a hydro-thermodynamic feedback to summer melt. *The Cryosphere* **9**, 197 – 215 (2015).

3. Moholdt, G., Nuth, C., Hagen, J. O. & Kohler, J. Recent elevation changes of Svalbard glaciers derived from ICESat laser altimetry. *Remote Sensing of Environment* **114**, 2756 – 2767 (2010).

4. Zemp, M. *et al.* Global glacier mass changes and their contributions to sea-level rise from 1961 to 2016. *Nature* **568**, 382 – 386 (2019).

5. Wouters, B., Chambers, D. & Schrama, E. J. O. GRACE observes small-scale mass loss in Greenland. *Geophysical Research Letters* **35**, L20501 (2008).

6. Mèmin, A., Rogister, Y., Hinderer, J., Omang, O. C. & Luck, B. Global Glacier Mass Loss During the GRACE Satellite Mission (2002-2016). *Geophysical Journal International* **184**, 1119 – 1130 (2011).

7. Gardner, A. S. *et al.* A reconciled estimate of glacier contributions to sea level rise: 2003 to 2009. *Science* **340**, 852 – 857 (2013).

8. Jacob, T., Wahr, J., Pfeffer, W. T. & Swenson, S. Recent contributions of glaciers and ice caps to sea level rise. *Nature* **514**, 514 – 518 (2012).

9. Matsuo, K. & Heiki, K. Current Ice Loss in Small Glacier Systems of the Arctic Islands (Iceland, Svalbard, and the Russian High Arctic) from Satellite Gravimetry. *Terrestrial, Atmospheric and Oceanic sciences journal* **24**, 657 – 670 (2013).

10. Wouters, B., Gardner, A. & Moholdt, G. Global Glacier Mass Loss During the GRACE Satellite Mission (2002-2016). *Frontiers in Earth Sicence* **7**, 11 p. (2019).

11. Aas, K. S. *et al.* The climatic mass balance of Svalbard glaciers: a 10-year simulation with a coupled atmosphere–glacier mass balance model. *The Cryosphere* **10**, 1089 – 1104 (2016).

12. Lang, C., Fettweis, X. & Erpicum, M. Stable climate and surface mass balance in Svalbard over 1979–2013 despite the Arctic warming. *The Cryosphere* **9**, 83 – 101 (2015).

13. Möller, M. & Kohler, J. Differing Climatic Mass Balance Evolution Across Svalbard Glacier Regions Over 1900–2010. *Frontiers in Earth Sicence* **6**, 20 p. (2018).

14. Østby, T. I. *et al.* Diagnosing the decline in climatic mass balance of glaciers in Svalbard over 1957–2014. *The Cryosphere* **11** (2017).

15. Van Pelt, W. *et al.* A long-term dataset of climatic mass balance, snow conditions, and runoff in Svalbard (1957–2018). *The Cryosphere* **13**, 2259 – 2280 (2019).

16. Schuler, T. V. *et al.* Reconciling Svalbard Glacier Mass Balance. *Frontiers in Earth Science* **8**, 16 pp. (2020).
